# A Systematic Review of the Evidence for Impaired Cognitive Theory of Mind in Maltreated Children

**DOI:** 10.3389/fpsyt.2015.00108

**Published:** 2015-07-28

**Authors:** Xavier Benarous, Jean-Marc Guilé, Angèle Consoli, David Cohen

**Affiliations:** ^1^Department of Child and Adolescent Psychiatry, Hôpital Pitié-Salpêtrière, Paris, France; ^2^Groupe de Recherches sur l’Analyse Multimodale de la Fonction Cérébrale, INSERM U1105, CHU and Université Picardie Jules Verne, Amiens, France; ^3^Paris-Sud Innovation Group in Adolescent Mental Health, INSERM U669, Maison de Solenn, Paris, France; ^4^UMR 7222, Institute for Intelligent Systems and Robotics, Université Pierre et Marie Curie, Paris, France

**Keywords:** theory of mind, false-belief, perspective-taking, attribution biases, child abuse, maltreatment, systematic review, social cognition

## Abstract

Compared to the large number of studies exploring difficulties in emotion recognition in maltreated children, few (*N* = 12) have explored the cognitive aspect of theory of mind (ToM), i.e., the ability to understand others’ thoughts and intentions. A systematic review of these studies shows inconsistent results regarding cognitive ToM tasks. Youths with a history of maltreatment are more likely to fail at false-belief tasks (*N* = 2). However, results are less conclusive regarding other tasks (perspective-taking tasks, *N* = 4; and hostile attribution tasks, *N* = 7). Additionally, only one study controlled for potential psychopathology. Measures of psychopathology and other cognitive abilities, in addition to ToM, are required to establish a specific association between maltreatment and the cognitive dimension of ToM.

## Introduction

In the past few decades, impairments in socio-emotional skills and empathy in children exposed to maltreatment have been recognized with the recent focus on individuals with callous-unemotional traits (1). The association has been claimed to exist even in the absence of psychopathology (2, 3). It has been suggested that a poor level of social understanding would partly explain the problematic peer relationships observed in maltreated children that lead to behavioral problems and peer rejection (3). The successful identification of the mechanisms underlying poor social abilities in maltreated children is essential to design specific interventions for this population. In this study, we explore the possible implications of one specific dimension of social cognition, the cognitive aspect of theory of mind (ToM), in the difficulties reported in maltreated children.

Theory of mind is defined as the awareness that others have a mind with various mental states (e.g., beliefs, desires, imagination, and emotion) that may differ from one’s own (4). It is one of the subcomponents of social cognition, i.e., the set of mental operations that underlie the ability to interact and communicate in accordance with social norms, values, and expectations (5). ToM is closely related to the development of empathy, the intuitive access to others’ subjective experiences (6). It denotes the capacity to understand others’ intentions and experience their feelings (6). Considering the importance of empathy in individuals’ ability to interpret socially relevant information, individual differences in ToM have important implications for social communication abilities (7, 8).

Theory of mind appears around age 4 in typically developing children and progresses from simple to more complex forms (5, 6, 9, 10). Infants’ early social behaviors rely on procedural empathy, an innate, non-verbal capacity to resonate with others’ emotional states (6). Procedural empathy encompasses three processes: (i) sensorimotor resonance and imitation, which are present in neonates (11); (ii) emotional mimicry (sympathy), when the subjectively experienced emotion is similar to the one observed in others (12); (iii) empathetic concern resulting from the attachment system, which develops between the infant and his/her primary caregivers (5). These precursors of ToM provide the bases for non-verbal communication between the child and his caregiver, which involve synchrony and reciprocity (13). Joint attention is present in children at 3 months and facilitates the ability to share a common point of reference, and then make inferences about others’ behaviors (5, 10). To predict and explain others’ behavior also implies the development of perspective-taking skills (e.g., the capacity to represent another person’s visual perspective) (12, 14, 15). In the second and third years, the ability to communicate with others firmly increases through the capacity to label emotions and concepts. Before the full development of ToM, language emergence paves the way for the development of a higher form of empathy – semantic empathy – that depends on verbal thought (16, 17). It involves different aptitudes: self-awareness (i.e., our own mental states are distinct from those of others) (18), the production of internal state words (19, 20), and the development of more general advances in symbolic maturity (21). More sophisticated interpersonal negotiation strategies continue to improve during adolescence and early adulthood. Biographical empathy, the capacity to bridge with others’ experiences, emerges later in life and corresponds to the interweaving of personal experience with feelings and words (6). Compared to procedural empathy, semantic and autobiographic forms of empathy may be more sensitive to childhood experience.

Empirical studies have noted that ToM development is strongly affected by non-heritable and environmental factors, such as parental practices (22–26), parental conversational elaboration (22), or the presence of siblings (27, 28). For example, Pavarini et al. found that ToM ability emerges earlier in families where: (i) children are considered to be intentional agents in verbal exchanges (e.g., using the second personal pronoun); (ii) mental states language is used (e.g., pointing out the causes and consequences of intentions, desires, or beliefs); and (iii) children are exposed to a wide range of emotions. As maltreating families are frequently characterized by disorganized interaction and poor parenting (29, 30), it would be expected that ToM difficulties occur in children from these families.

A growing literature reports impairments in social abilities in maltreated children compared to non-maltreated children (3). However, the mechanisms underpinning such difficulties remain incompletely understood. Despite a large number of studies focusing on maltreated children’s difficulties recognizing others’ emotions, few studies have explored their capacity to infer others’ thoughts or intentions (3, 31). There is indeed growing evidence that the recognition of another’s emotions and thoughts, respectively, referred to as affective ToM (or “hot” ToM) and cognitive ToM (or “cold” ToM), are related but distinct domains (32–35). Such capacities would depend in part on separate anatomical substrates (36, 37). Affective and cognitive ToM may be independently impaired in individuals with psychopathology (1, 6, 36, 38) or neurologic diseases (39). To distinguish cognitive and affective ToM, help to map specific domains of cognitive development altered in maltreated children. In doing so, it would provide possible mechanisms by which these children are at higher risk of developing severe behavioral disturbances and psychopathology (3). In particular, a better knowledge of these mediators is a major prerequisite for developing preventive interventions (30).

Our aim in this paper is to conduct a critical review examining the objective evidence for a specific impairment in cognitive ToM in maltreated children.

## Materials and Methods

**Figure 1:**
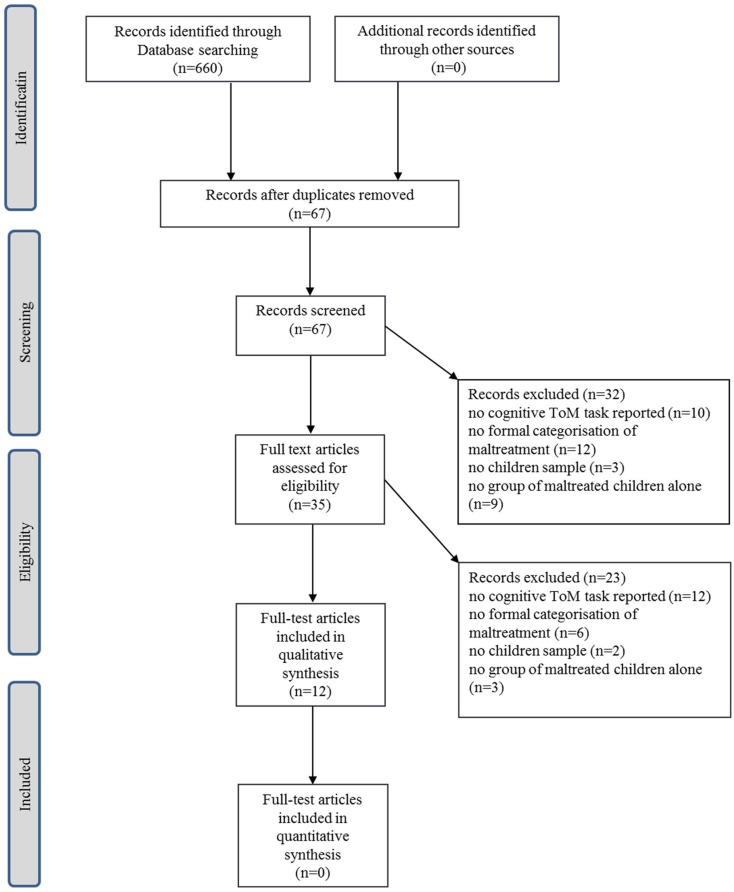
**PRISMA diagram of screening process and study selection**.

A systematic review was conducted, following the recommendations outlined in the PRISMA guide (40). The distinction between the behavioral tasks measuring cognitive and affective ToM, proposed by Henry et al. (33) and Brothers and Ring (32), were used to determine keywords. Cognitive ToM tasks are those requiring an understanding of others’ beliefs, thoughts, or intentions; affective ToM tasks typically ask participants to describe a protagonist’s emotion. The following terms were used in the literature search: *social cognition* or *empathy* or *theory of mind* or *false-belief* or *social perception* or *social knowledge* or *attributional bias* and *child abuse* or *child maltreatment* or *early trauma* or *early adverse events* or *sexual abuse* or *physical abuse* or *emotional abuse* or *neglect*. Three online databases – PsychInfo, PubMed, and Scopus – were used to identify relevant records on the basis of these search criteria. All articles published between 1980 and July 2014 were considered for inclusion in this review. All records resulting from the initial search were screened according to *a priori* inclusion criteria (Figure 1). These criteria were:

- Use of a task to measure cognitive ToM (i.e., attribution of intentions or beliefs). By contrast, studies where only emotional ToM was measured (i.e., attribution of emotion or feelings) were not selected. We used the distinction proposed by Henry et al. (33) and Luke and Banerjee (3) between ToM tasks measuring cognitive ToM, affective ToM, or both.
- Assessment of at least one of the following widely recognized forms of childhood maltreatment (41): physical abuse, sexual abuse, emotional abuse, emotional negligence, or physical negligence. Moreover, studies that explored the broader area of parenting style in relation to ToM as well as other forms of childhood adversity, such as parental loss or separation, bullying, or a wide range of early stressful life events, were considered to be outside the scope of this review.
- Studies conducted in children or in which a subgroup of children was analyzed. Studies were not selected if the results were only presented for childhood and adulthood together. Studies in adolescents only (older than 13 years old) were not selected.
- Presence of a comparison group in the study design. We did not retain studies in which maltreatment were explored as a covariate, without presenting data separately for individuals with and without a history of maltreatment.

The relevant findings were extracted from the final set of papers by the first two authors. To clarify the presentation of results, we used the distinction proposed by Luke and Banerjee (3); i.e., results are separated into three main sections according to the tasks used: the false-belief task, the perspective-taking task, and measures of attributional bias. The two others tasks proposed by Luke and Banerjee (3) (i.e., *emotion understanding* and *emotion recognition)* were not explored here, as they refer to the affective, not the cognitive, aspect of ToM.

## Results

In total, 12 studies were included in this review. Sample characteristics for each study are shown in Tables 1–3. It was not possible to perform any meta-analysis of the data generated by the current review due to the small number of studies and lack of task replication across studies. Therefore, a descriptive synthesis of the available data is provided according to the tasks involved.

**Table 1:** **Details of studies included in the review for false-belief tasks**.

Authors (country)	*N*	% female	M age (range)	Sample recruited	Control recruited	Study design	Measure of abuse	Type of child abuse	Age at which abuse occurred/identity of the abuser	Tasks used	Measure of psychopathology/comorbidity rate
Cicchetti, et al. (42) (USA)	203 (M)	35% (M)	6.0 (3–8) (M)	From child protective service agency, living in their own family	From families receiving public assistance for the low-SES group via hospital birth records and fliers posted in neighborhood for the middle-SES group	Cross-sectional	MCS	PA/SA/EA/N	A/A not reported	Two unexpected-content task	NA/NA
	315 (NM)										
	Low-SES: 143	45% (NM)	6.2 (3–9) (NM)								
	Middle-SES: 172										
Pears and Fisher (43) (USA)	60 (M)	52% (M)	4.3 (3–5) (M)	From child protective service agency, all children entering new foster placements for maltreatment	From neighborhood via targeted mailings and advertisements, matched on age, SES, living with their biological families without previous involvement in child welfare services	Cross-sectional	Administrative report	PA/SA/EA/N	NA/NA	Combined measure including three discrepant belief tasks	NA/NA
	31 (NM)	55% (NM)	4.4 (3–5) (NM)								

*A, available; NA, no available; M, maltreated; NM, no maltreated; MCS, Maltreatment Classification System by Barnett; 1Q, intelligence quotient; SES, socioeconomic status*.

**Table 2:** **Details of studies included in the review for perspective-taking tasks**.

Authors (country)	*N*	% Female	M age (range)	Sample recruited	Control recruited	Study design	Measure of abuse	Type of child abuse	Age at which abuse occurred/identity of the abuser	Tasks used	Measure of psychopathology/comorbidity rate
Barahal et al. (44) (USA)	17 (M)	72% (M)	7.5 (6–8) (M)	From child protective service agency in Colorado and in Denver, living in their own family	From enrollment list of a summer camp, which was supported by the same social service agency; matched on social class, family configuration	Cross-sectional	Administrative report	PA/SA	NA/A	Boy–dog–tree test	NA/NA
	16 (NM)	25% (NM)	7.5 (6–8) (NM)								
Walker and Downey (45) (USA)	47 (M)	53% (M)	9.7 (7–15) (total)	From child protective service agency and local psychiatric facilities	From local psychiatric facilities	Cross-sectional	Administrative report	PA/NE	NA/NA	Word-communication task: selecting best clues for imaginary partner to pass word task	NA/NA
	55 (NM)	44% (NM)									
Pears and Fisher (43) (USA)	60 (M)	52% (M)	4.3 (3–5) (M)	From child protective service agency, all children entering new foster placements for maltreatment	From neighborhood via targeted mailings and advertisements, matched on age, SES, living with their biological families without previous involvement in child welfare services	Cross-sectional	Administrative report	PA/SA/EA/N	NA/NA	Combined measure including spatial perspective-taking task	NA/NA
	31(NM)	55% (NM)	4.4 (3–5) (NM)								
Burack et al. (46) (USA)	26 (M)	12% (M)	10.2 (7–12) (M)	From child protective service agency, living in their own family, in foster care, in group homes and from a program for youths with behavioral and emotional problems	From local school via targeted mailings, matched on age, gender, ethnic status, SES, IQ, marital status; screened negative for a history of abuse	Cross-sectional	Administrative report, Parent–Child Relationship Checklist	PA/SA/N	NA/NA	Chandler’s Bystander Cartoons test	CBCL-TRF/NA
	26 (NM)	12% (NM)	10.3 (7–12) (NM)								

*A, available; CBCL, Child Behavior Check List; NA, no available; M, maltreated; NM, no maltreated; IQ, intelligence quotient; SES, socioeconomic status*.

**Table 3:** **Details of studies included in the review for hostile attribution tasks**.

Authors (country)	*N*	% Female	M age (range)	Sample recruited	Control recruited	Study design	Measure of abuse	Type of child abuse	Age at which abuse occurred/identity of the abuser	Tasks used	Measure of psychopathology/comorbidity rate
Downey and Walker (47) (USA)	36 (M)	46% (M)	9.6 (8–10) (M)	From child protective service agency and local psychiatric facilities	From local psychiatric facilities	Cross-sectional	Administrative report	PA/N	NA/NA	Assigning intent to story characters in cartoons story	CBCL/NA
	47 (NM)	41% (NM)	10.0 (8–10) (NM)								
Dodge et al. (48) (USA)	46 (M)	47% (total)	4.0 at baseline	From kindergarten pre-registration in April 1987 in three different regions		Longitudinal, multicentric	Score likelihood of physical harm	PA	NA/NA	Assigning intent to story characters in cartoons story	CBCL/NA
	258 (NM)										
Price and Glad (49) (USA)	44 (M)	52% (M)	6.5 (4–6) (total)	From child protective service agency, initially contacted by mail	From neighborhood via targeted mailings and advertisements, with no prior contact with any social service agencies	Cross-sectional	MCS	PA/N	NA/NA	Assigning intent to story characters in cartoons story	NA/NA
	56 (NM)	50% (NM)									
Ayoub et al. (50) (USA)	27 (M)	32% (total)	4.0 (1.8–6.1) (M)	From child protective service agency, living in their own family in three centers in an urban, low income neighborhood	From child protective service agency, screened negative for a history of abuse, matched on ethnic status and SES	Cross-sectional	MCS	PA/SA/EA/N	A/A not reported	The Mean and Nice Interaction Scales: retelling stories of mean and nice interactions	NA/NA
	26 (NM)		3.2 (1.8–6.1) (NM)								
Teisl and Cicchetti (51) (USA)	167 (M)	38% (M)	8.2 (6–12) (M)	From two cohorts of children followed by protective service agency, initially contacted by mail	From neighborhood via targeted mailings and advertisements, screened negative for a history of abuse	Cross-sectional	MCS	PA/SA/EA/N	NA/NA	Assigning intent to story characters with a series of videotaped vignettes	NA/NA
	100 (NM)	45% (NM)	8.6 (6–12) (NM)								
Sabourin Ward and Haskett (52) (USA)	98 (M)	50% (total)	7.3 (5–10) (total)	From child protective service agency, initially contacted by mail	From neighborhood via targeted mailings and advertisements, screened negative for a history of abuse, matched on demographic factors	Cross-sectional	Administrative report	PA	NA/NA	Home Interview with children: assigning intent to story characters in cartoons story	NA/NA
	77 (NM)										
Keil and Price (53) (USA)	100 (M)	51% (total)	6.5 (5–8) (total)	From child protective service agency, living in their own family	From neighborhood via targeted mailings and advertisements, with no prior contact with any social service agencies	Cross-sectional	MCS	PA/N	A/A reported	Assigning intent to story characters in cartoons story	NA/NA
	88 (NM)										

*A, Available; CBCL, Child Behavior Check List; NA, no available; M, maltreated; NM, no maltreated; MCS, Maltreatment Classification System by Barnett; IQ, intelligence quotient; SES, socioeconomic status*.

### False-belief tasks

The classic “false-belief task” was initially a story acted out with dolls and props in the Sally and Anne Test (4). A first-order task that requires understanding of another person’s mental state is passed by 3–4 years of age for a typically developing child (9). Second-order false-belief tasks involve understanding what two people think sequentially, for example, “Pierre thinks what Paul thinks”; such tasks are usually passed by ~6 years of age. Control tasks include true belief trials, where the reality of a situation can be solved without any thought inference. Language development, age, and socioeconomic status (SES) have been empirically linked to children’s understanding of false-beliefs (33). Modality of task presentation differed between studies: one study used video to present the story while another used cartoons and verbal narrative.

Two studies compared false-beliefs tasks between maltreated and non-maltreated children. Findings from these two studies are consistent; children with a history of maltreatment showed poorer performance on false-belief tasks compared with their peers. Cicchetti et al. (42) showed the performance on two different unexpected-content tasks in a group of 4–8-year-olds, maltreated and non-maltreated children. They found that physically, sexually, or emotionally abused children had lower scores than non-maltreated peers. This would especially concern those youths for whom maltreatment had begun the earliest. Pears and Fisher (43) found that success rate at a discrepant belief tasks were lower among abused or neglected 3–5-year-olds compared to non-maltreated children. However, in this study, the principal outcome was a combined measure of four different tasks (i.e., a false-belief task, a perspective-taking task, a desire understanding task, and an appearance–reality task) (54). In these two studies, results persisted after controlling for chronological age, intelligence quotient (IQ) (WPPSI-R score), and SES.

### Perspective-taking tasks

Perspective-taking tests require participants to adopt a third person’s perspective while making spatial judgments (i.e., visual perspective taking) or retelling a story from another’s point of view (i.e., conceptual perspective-taking) (12, 55).

Only one study explored visual perspective-taking in maltreated children (43). Pears and Fisher (43) found that children in foster care had difficulties performing ToM tasks, after controlling for age, intelligence, and executive function. As the main outcome was a combined measure (including a false-belief task, a two-level perspective-taking task, a desire understanding task and an understanding of appearance–reality task), it was difficult to discern which specific domains were impeded.

Two out of three studies showed poor conceptual perspective-taking skills in maltreated children who were asked to retell stories from a different point of view. Barahal et al. (44) explored this ability among a group of physically abused 6–8-year-olds. Abused children, compared to non-abused peers, more frequently continued to believe that others would describe stories similar to their own. Even after they knew that important story components had been removed in a boy–dog–tree task, 35% of the abused children maintained persistently egocentric views. This finding was no longer significant with IQ controlled. Burack et al. (46) found a higher level of egocentrism among abused or neglected 7–12-year-olds compared to controls using the Chandler’s Bystander Cartoons Test. This association was independent of self-esteem and externalizing and internalizing symptoms as measured by the Child Behavior Check List (CBCL). In contrast, Walker and Downey (45) sample of neglected 9-year-olds, compared to non-maltreated peers, showed impairments in perspective-taking tasks; however, performances of the two groups were comparable after controlling for gender, parental psychopathology, IQ (WISC-R), and age. The fact that the control group was partly recruited from local inpatient and outpatient facilities makes it difficult to interpret the data.

### Social attribution tasks

Except for one study (50), social attributions have been assessed using a series of vignettes adopted from the work of Crick and Dodge (56) on social information processing in children. This task presents hypothetical situations depicting a child experiencing some type of negative outcome in a social situation where the intentions of the other person in the story are ambiguous. In these types of situations, children must rely on their internal mental representations to guide their interpretation of the other’s intentions.

Three out of seven studies found a hostile attribution bias in maltreated children when asked to attribute intent for negative acts in response to ambiguous scenarios. Dodge et al. (57) showed that physically abused children had more encoding errors, more hostile attributional biases, more accessing of aggressive response to peers, and more positive evaluation of the outcomes of aggression compared to non-abused children. As aggressive behaviors were measured through the CBCL teacher report in the same sample 4 years later, a mediation model was built in which difficulties in social attribution explained 33% of the effect of a history of abuse on the onset of aggressive behavior (48). The authors conceded that the lack of evaluation of IQ reduced the scope of the study. Ayoub et al. (50) explored the ability to retell complex stories upon different emotional contexts in abused and neglected 3–6-year-old children. They found that the maltreated children have more difficulty representing positive social interactions, although they were as good as non-maltreated peers at retelling stories about negative interactions. The authors suggested that maltreated children’s abilities to think with cognitive complexity is sustained but applied differentially to situations based on emotional valence. Keil and Price (53) found that 5–8-year-old children who had been neglected and physically abused were more likely to attribute hostile intents in response to videotaped vignettes about peer provocation compared to non-maltreated peers.

Three studies provided mixed evidence regarding hostile attributions in maltreated children. Price and Glad (49) found that 4–6-year-old boys who had been physically abused or who witnessed domestic violence were more likely to attribute hostile intentions to a variety of figures, including their parents, an unfamiliar teacher, their best friend, and unfamiliar peers. The effect increased with the frequency of physical abuse. However, this result was not found for girls. The authors proposed that physically abused girls who participated in this investigation experienced less frequent and severe forms of physical abuse than did the boys; an alternative hypothesis is that boys are more likely to be involved in social interactions that contribute to the development and maintenance of hostile attributions after abuse. Mediation analysis found these children’s hostile attributions of their mothers mediated the relationship between physical abuse and children’s hostile attributions of unfamiliar peers. Teisl and Cicchetti’s (51) sample of physically abused 6–12-year-olds gave no more hostile attributions to ambiguous vignettes than did non-maltreated children. Even if overall group differences were not significant, authors noted that physically abused children were more likely to incorrectly interpret prosocial and accidental scenarios as hostile and were more likely to have aggressive responses to provocation (after controlling for age, gender, and race/ethnicity). Sabourin Ward and Haskett (52) found no difference between physically abused 5–10-year-olds and non-maltreated children in their understanding of interpersonal situations. Using person-centered method, authors identified two distinct profiles among youths with little prosocial behavior. Youths with little prosocial and little maladjusted behavior, e.g., rarely rejected by peers (“Hanging in There” group), more frequently reported hostile attributions of intent in response to vignettes than those with little prosocial behavior and high social maladjustment (“Social Difficulties” group). Walker and Downey (45) sample of physically abused or neglected 7–14-year-olds did not face more difficulties to interpret interpersonal relationships compared to peers.

## Discussion

### Study limitations

There were several limitations in the available data that preclude us from drawing firm conclusions with regard to the impact of maltreatment on cognitive ToM in children.

The first limitation concerns the measure of maltreatment. Indeed, the evaluation of child maltreatment has been regarded as an important issue in previous reviews in Ref. (30, 41, 58). Maltreatment is defined as any acts of commission or omission by a parent or other caregiver that result in harm, potential for harm, or threat of harm to a child (41). Four forms of maltreatment are widely recognized: physical abuse, sexual abuse, psychological (or emotional) abuse, and neglect. Despite the development of consensual definitions and standardized approaches to explore and quantify maltreatment, some types of abuse, especially neglect and emotional abuse, remain difficult to evaluate (30, 42). In our review, five studies used the Maltreatment Classification System proposed by Barnett (42, 49–51, 53, 59). In four studies, the severity and frequency of the abuse and the identity of the abuser were explored, but this information was only reported in two studies (44, 53), likely because the sample sizes were too low to reach statistical significance in most of the analyses. It should also be noted that most studies included no mention of whether assessors were blind to children’s maltreatment status or not.

The second limitation is the lack of reporting on psychopathological symptoms in the samples studied. Only one study reported psychiatric comorbidities and controlled for psychopathologic score when examining the relationship between maltreatment and cognitive ToM (46). Altered social cognitions have been reported in children suffering from internalized and externalized psychiatric disorders (1, 60, 61). Considering the high prevalence of psychiatric comorbidities in maltreated children (30, 31, 34, 62), extreme caution is required in interpreting the results of studies without any measure of psychopathology. The question of whether cognitive ToM impairment occurs in maltreated children, even in the absence of psychopathology, has significant implications for therapeutic approaches. If this assumption is confirmed, interventions aiming at promoting social abilities in maltreated children by facilitating cognitive ToM abilities should not only focus on children with psychiatric symptoms. To answer this question, psychopathology, in addition to cognitive skills, should be assessed and reported in future research. Then, follow-up studies could help to determine to what extend deficits in cognitive ToM in maltreated children led to the development of overt cognitive, affective, or behavioral disturbance found in psychopathology.

The third limitation is the cross-sectional design of most of the studies reviewed. Indeed, the relationship emphasized in cross-sectional studies could be interpreted in the opposite direction, such as, children with poor cognitive ToM may be more frequently involved in intra-familial abuse (63). For example, in a large community-based sample, Shakoor et al. (63) showed that poor ToM predicted becoming a victim of bullying in early adolescence. In the same way, Sullivan and Knutson (64) noted that an increased prevalence of maltreatment in children with disabilities was associated with lower social cognition abilities. In this study, the risk of enduring all types of maltreatment was four times higher in children with mental retardation, and five times higher in children with speech and language impairments. Other longitudinal studies would be useful to explore the direction of the relationship between cognitive ToM and maltreatment.

The fourth limitation is the wide disparity in the composition of control groups (i.e., non-maltreated children) used in the studies. Maltreated children often experience multiple adversities, including poverty, parental psychopathology, or parental unemployment, which correlate with lower level social-cognitive development (30). For example, language, which is an essential factor for the emergence of a ToM (17), is strongly associated with the SES of the families (65). When examining the specific impact of maltreatment on social-cognitive abilities, the control group should be as similar as possible to the maltreated group on all factors associated with ToM. In three studies from the current review, the control group was composed of children whose families did not have prior contact with social service agencies (43, 49, 53). By contrast, Ayoub et al. (50), Cicchetti et al. (42), and Barahal et al. (44) compared maltreated youth with children whose families were receiving public assistance but who screened negatively for maltreatment. In the latter case, it may be more difficult to find a significant difference between the maltreated and control groups. Therefore, the positive findings reported by Pears and Fisher (43), Price and Glad (49), and Keil and Price (53) should be interpreted with care. However, the majority of the studies discussed here matched their maltreatment group and control groups on other factors known to affect social understanding, such as age and SES (46). Burack et al. (46) also controlled for children’s IQ. As an alternative, Cicchetti et al. (42) proposed a stratified sampling between three groups to independently explore the effect of maltreatment and SES on performance of a false-belief task.

### Summary of the main results

The poorer performance in false-belief tasks in maltreated children was consistent between studies (42, 43). However, results were less consensual regarding perspective-taking tasks. Only one study found specific difficulties in perspective-taking tests in maltreated children (46). The difference showed by Barahal et al. (44) between maltreated and non-maltreated youths did not persist after controlling for IQ, and one study found no difference (45). Findings regarding hostile attribution bias in maltreated children were also mixed. Dodge et al. (48) and Keil and Price (53) found a significant difference in the level of hostile attribution between maltreated and non-maltreated children, whereas Price and Glad (49), Teisl and Cicchetti (51), and Sabourin Ward and Haskett (52) did not report such difference. Two studies were difficult to interpret because of the task used to examine attributional bias (50) and the severity of the control group (47).

### Interpretation

The current findings suggest that there is limited available evidence of poorer cognitive ToM in child victims of maltreatment. In addition, we noted a large divergence in findings depending on how cognitive ToM was measured.

In comparison with non-maltreated children, youths with a history of maltreatment showed more difficulties with false-belief tasks. Some authors concluded that difficulties in cognitive ToM were independent of other deficits in cognitive functions; however, few studies controlled for the potential impact of other cognitive functions on their results. Controlling for cognitive deficits is important, as previous reports have noted that false-belief tasks involve other cognitive abilities (9) such as working memory, joint attention, or executive function (10). Therefore, an independent effect of maltreatment on the ability to understand other’s thoughts or intentions is consistent with the model of empathy proposed by Guile (6). In this model, the development of more elaborate forms of empathy continues throughout childhood and adolescence (i.e., “semantic” and “biographic” empathy) depending on the environmental context.

Discrepancies between studies exploring perspective-taking have been noted in our review. These discrepancies may be partly explained by the diversity in the tasks used and the age of the participants. First, differences in the level of inappropriate egocentric thinking between maltreated and non-maltreated youths was more marked in adolescents than in school-aged children according to Burack et al. (46). The lack of significant results in studies exploring conceptual perspective-taking tasks could be explained by the young age of the sample studied. Future studies should explore these results in distinct child and adolescent samples. Second, from our findings, it is still unclear whether spatial perspective-taking is impaired in maltreated children, considering the lack of specific measurement in the study of Pears and Fisher (43). This final assumption is supported by growing empirical studies exploring the relationship between visuo-spatial mechanisms and empathy in healthy children. Thirioux et al. (12) suggested that the cognitive component of empathy refers to a controlled process whereby individuals understand the mental states of others while adopting their psychological viewpoint. Therefore, attribution of the other’s experience to oneself requires, among other skills, visual perspective-change by the child to enable mental imagery in a mirror-like manner (i.e., heterocentered visuo-spatial) (12). Further studies should explore whether poor ability to develop visuo-spatial perspective may be one process by which empathy is disturbed in maltreated children.

Studies where social attribution bias in maltreated children was examined showed heterogeneous results. Our review supports a gender difference in the effect of maltreatment on hostile attribution bias that may explain such discrepancies. Price and Glad (49) found a difference in hostile attribution only in boys, but not in girls, with a history of maltreatment. Such difference is consistent with the works of Teisl and Cicchetti (51) who reported a higher level of hostile attribution in maltreated boys compared to girls. Moreover, the subgroup of maltreated children with the highest level of hostile attribution in the cluster analysis proposed by Sabourin Ward and Haskett (52) also had the greatest proportion of males compared to other subgroups. Although some studies reported outcomes for each gender separately, this was not the case for all papers. This circumstance may be a source of bias for three main reasons. (i) Findings indicate that some forms of abuse may be more common among women than men (41, 66), and perhaps only specific type of abuse have consequences for ToM (49). (ii) Abuse could have greater association with psychopathology and social impact according to gender (49). For example, compared to boys, disruptive disorders in girls with a history of physical abuse are less frequent but are associated with poorer outcome (e.g., more functional impairment and social consequences) (66). (iii) Development of ToM could differ between girls and boys, for example, on the age required to pass specific ToM tasks (33). In this context, future studies should take into account a possible gender difference in exploring the effect of maltreatment on hostile attribution.

Moreover, interpretation of studies concerning social attribution in maltreated children should also take into consideration that such tasks are strongly related to emotional understanding ability, i.e., the affective component of ToM. Indeed, recognition of affective states emerges before the child begins to understand their causes, and development of the latter may be more susceptible to the deleterious effects of maltreatment (67). Teisl and Cicchetti (51) showed that the association between hostile attribution and behavior problems in physically abused children was no longer significant when controlling for emotion regulation. Difficulties in emotion recognition and understanding observed in child victims of abuse or maltreatment may affect social information processing and contribute to social attribution bias, compared to children in non-maltreated context (3). For example, Pollak et al. (68) found that physically abused children showed a bias toward anger when reacting to ambiguous stimuli. Co-occurring deficits in emotion regulation and in ToM observed in maltreated children families may operate through poor child–caregiver interactions (31, 69). For example, Meins et al. (23) suggested that the development of a secure attachment facilitates the mentalizing ability (i.e., the process of making sense of one’s own and other’s mental states), which plays a role both in the capacity for understanding others’ intents and feelings but also in understanding and regulating one’s emotion. Therefore, disorganized or insecure attachment may impede the development of the mentalizing skill involved in both cognitive and affective ToM (70–72).

### Clinical implications and direction for future research

We have highlighted the importance of considering multiple domains of development in exploring cognitive ToM in maltreated children and of controlling for psychopathology and contributing cognitive abilities (e.g., working memory, joint attention, or executive function). Only then, could we precisely answer whether there are difficulties in cognitive ToM abilities in abused children, and, if so, how these difficulties are integrated to the more general social-cognitive impairments found in these children. Studies are needed to map the dynamic interactions between social-cognitive development and other cognitive abilities from early infancy to late adolescence. As illustrated with the model of empathy that distinguishes procedural, semantic, and autobiographic dimensions (6), the emergence of social cognition is affected by factors occurring at different sensitive periods throughout child development. Minor impairments in social-cognitive abilities may only become apparent in adolescence. It is therefore important that studies could be conducted in youth using standardized cognitive tasks across various age ranges.

Studies with large sample sizes and repeated measures of cognitive abilities (e.g., ToM and social attribution), psychopathology, and general functioning (including social performance) collected from infancy to late adolescence would be of great value. Based on these data, path analysis could be performed to test whether, and to what extent, cognitive difficulties mediate or moderate the risk for poor outcomes (i.e., psychopathology and social adaptation) in maltreated youth.

The resulting findings may, in turn, support the development and evaluation of treatment approaches for abused children that target social and emotional processing. First, studies in maltreated youth could help determine the feasibility and benefits of specific therapeutic interventions on social cognition (e.g., cognitive remediation). Second, future research should also focus on the environmental (e.g., foster care conditions) and therapeutic interventions that might moderate the long-term effects of maltreatment on social cognition (30, 41, 58). This is particularly important as poorly thought out social interventions (e.g., multiple changes in the child’s placement and early attempts at reunifying the child with his/her biological family) increase the risk of developing poor psychosocial outcomes (41).

## Conclusion

Considering the limited available data, it was not possible to draw any firm conclusions about the association between childhood maltreatment and direct impairments in the cognitive component of ToM. Results were inconsistent with variations according to the tasks used; however, preliminary evidence supports difficulties in performance on false-beliefs tasks among child victims of maltreatment. Moreover, many studies did not control for psychopathology, making the results more difficult to interpret. Models linking cognitive ToM and emotion regulation via possible mediators or moderators (e.g., mentalization or attachment) need to be tested in future research, preferably using prospective designs.

## Author Contributions

Substantial contributions to the conception and design of the work: XB, J-MG, and DC. Substantial contributions to the acquisition, analysis, or interpretation of data: XB and AC. Drafting the work or revising it critically for important intellectual content: XB, AC, J-MG, and DC. Final approval of the version to be published: J-MG, AC, and DC. Agreement to be accountable for all aspects of the work in ensuring that questions related to the accuracy or integrity of any part of the work are appropriately investigated and resolved: XB, AC, J-MG, and DC.

## Conflict of Interest Statement

The research was conducted in the absence of any commercial or financial relationships that could be construed as a potential conflict of interest.
